# Global burden and trends of rotavirus infection-associated deaths from 1990 to 2019: an observational trend study

**DOI:** 10.1186/s12985-022-01898-9

**Published:** 2022-10-20

**Authors:** Yuxia Du, Can Chen, Xiaobao Zhang, Danying Yan, Daixi Jiang, Xiaoxiao Liu, Mengya Yang, Cheng Ding, Lei Lan, Robert Hecht, Changtai Zhu, Shigui Yang

**Affiliations:** 1 Department of Public Health, State Key Laboratory for Diagnosis and Treatment of Infectious Diseases, National Clinical Research Center for Infectious Diseases, Collaborative Innovation Center for Diagnosis and Treatment of Infectious Diseases, The First Affiliated Hospital, Zhejiang University School of Medicine, 79 Qingchun Road, Hangzhou, China; 2 Department of Epidemiology of Microbial Diseases, Yale School of Public Health, New Haven, CT 06520 USA; 3 Department of Laboratory Medicine, Shanghai Jiao Tong University Affiliated Sixth People’s Hospital, Shanghai, China

**Keywords:** Rotavirus, Global burden, Global trends, Joinpoint regression model

## Abstract

**Background:**

Rotavirus is the leading global pathogen of diarrhea-associated mortality and poses a great threat to public health in all age groups. This study aimed to explore the global burden and 30-year change patterns of rotavirus infection-associated deaths.

**Methods:**

Based on the Global Burden of Disease 2019 Study (GBD 2019), we analyzed the age-standardized death rate (ASDR) of rotavirus infection by sex, geographical region, and sociodemographic index (SDI) from 1990 to 2019. A Joinpoint regression model was used to analyze the global trends in rotavirus infection over the 30 years, SaTScan software was used to detect the spatial and temporal aggregations, and a generalized linear model to explore the relationship between sociodemographic factors and death rates of rotavirus infection.

**Results:**

Globally, rotavirus infection was the leading cause of diarrheal deaths, accounting for 19.11% of deaths from diarrhea in 2019. Rotavirus caused a higher death burden in African, Oceanian, and South Asian countries in the past three decades. The ASDR of rotavirus declined from 11.39 (95% uncertainty interval [95% UI] 5.46–19.48) per 100,000 people in 1990 to 3.41 (95% UI 1.60–6.01) per 100,000 people in 2019, with an average annual percentage change (AAPC) (− 4.07%, *P* < 0.05). However, a significant uptrend was found in high-income North America (AAPC = 1.79%, *P* < 0.05). The death rate was the highest among children under 5 years worldwide. However, the death rates of elderly individuals over 70 years were higher than those of children under 5 years in 2019 among high, high-middle, middle, and low-middle SDI regions. Current health expenditure, gross domestic product per capita, and the number of physicians per 1000 people were significantly negatively correlated with death rates of rotavirus.

**Conclusions:**

Although the global trends in the rotavirus burden have decreased substantially over the past three decades, the burden of rotavirus remained high in Africa, Oceania, and South Asia. Children under 5 years and elderly individuals over 70 years were the populations most at risk for rotavirus infection-associated deaths, especially elderly individuals over 70 years in relatively high SDI regions. More attention should be paid to these areas and populations, and effective public health policies should be implemented in the future.

**Supplementary Information:**

The online version contains supplementary material available at 10.1186/s12985-022-01898-9.

## Introduction

In 2016, diarrheal diseases were the eighth leading cause of death among all ages and the fourth leading cause of death among children under 5 years [1, 2]. Rotavirus was the leading pathogen of death of diarrheal disease [1, 3–5], causing more than 258,000,000 (95% uncertainty interval [95% UI]: 193,000,000–341,000,000) infections and nearly 129,000 (95% UI 104,500–155,600) deaths among children under 5 years globally in 2016 [2]. To prevent rotavirus infection, the World Health Organization (WHO) first recommended including the rotavirus vaccine in national immunization programs in the European region and America in 2006. It was recommended in 2009 that the vaccine be extended to all regions worldwide [6, 7].

As the main pathogen of gastroenteritis, rotavirus infection and death occur in all countries worldwide, but mainly in developing countries [6]. In recent decades, some studies have reported reductions in rotavirus infections and deaths due to improvements in safe water, sanitation, and medical care, as well as advances in prevention and treatment, such as using rotavirus vaccines [8, 9]. However, the burden of rotavirus remained high, and rotavirus was still a highly prevalent cause of diarrhea worldwide [2]. In addition, several studies have found an upward trend in the burden of rotavirus among elderly individuals. In Europe, the European Rotavirus Network (EuroRotaNet) has found that the proportion of rotavirus infections among elderly individuals has increased in countries such as Germany and Finland [10]. In Australia, a study showed increased hospitalization rates among elderly individuals [11].

Although some studies have reported the burden of rotavirus in certain regions or countries, the global burden and epidemiological trends of rotavirus change during long-term epidemics have rarely been studied. In particular, the burden and trends of rotavirus among elderly individuals (adults 70 years and older) have not been systematically elucidated. In addition, different risk factors between regions, unbalanced medical and health resources, and differences in the sociodemographic index (SDI) might affect the death burden of rotavirus. However, there is no systematic study on this aspect. Thus, based on the latest data from the Global Burden of Disease 2019 Study (GBD 2019), we investigated the global burden and the change in the pattern of rotavirus-associated mortality to identify regions or populations with high or rising mortality. We provide insights into the formulation of targeted policies and actions to reduce the burden of rotavirus.

## Methods

### Data sources

We obtained data on the global burden of rotavirus from GBD 2019. Comprehensive and standardized assessments of the world’s health for 369 diseases, injuries, and impairments from 1990 to 2019 were available in GBD 2019, using consistent methods across countries to compare them. GBD 2019 included 204 countries and territories divided into 21 regions based on epidemiological similarities and geographical proximity and five groups according to SDI quintiles (low, low-middle, middle, high-middle, and high SDI). The SDI is a comprehensive indicator of economic growth, educational attainment, and fertility rate. This study extracted the number of deaths, death rates, and ASDRs of rotavirus infection with 95% UIs from 1990 to 2019, and stratified analyses were conducted by age, sex, location and year.

### Joinpoint regression model analysis

We used a joinpoint regression model to examine the temporal trend of ASDRs of rotavirus infection in different SDI regions from 1990 to 2019. The annual percentage changes (APCs) and average annual percentage changes (AAPCs) with 95% confidence intervals (CIs) were then calculated by fitting a regression line to the natural logarithm of the rates using the year as a regression variable. We used the Z test to assess whether an APC and an AAPC differed significantly from zero. If the APC and AAPC were significant (*P* < 0.05), they were considered to be increasing or decreasing; Otherwise, they were considered stable.

### Spatial and temporal aggregation analysis

We retrospectively identified clusters of rotavirus infection-associated deaths via spatial and temporal aggregation analysis using the statistical package SaTScan (version 9.5). Poisson distribution was selected for the probabilistic model, and a dynamic space–time two-dimensional cylindrical scanning window was established to scan time and country units. The expected number of deaths, logarithmic likelihood ratios (LLRs), and relative risks (RRs) were calculated in each scan window. LLRs were used to estimate the most likely clustering regions and then classify the clusters according to the estimated size of the LLRs. Monte Carlo simulation was used to evaluate statistical significance, and the significance level was set at *P* < 0.05.

### Generalized linear model (GLM)

We selected 7 covariates from 14 country-level sociodemographic covariates across 199 countries from 1990 to 2019, including the gross domestic product (GDP) per capita, current health expenditure (% of GDP), current health expenditure per person, percentage of the population aged 0–14, the number of physicians per 1000 people, the proportion of the urban population and the number of population. Several types of GLMs were used to fit each country-level sociodemographic covariate and death rates of rotavirus. According to the Akaike information criterion, the best fitting model of corresponding variables was determined to be a Gaussian distribution. The fitted model equation is as follows:

Log[E(Yt)] = *β0* + *β*(Current health expenditure (% of GDP)) + *β*(Log current health expenditure per person) + *β*(Percentage of the population aged 0–14) + *β*(Log GDP per capita) + *β*(Number of physicians per 1000) + *β*(Proportion of the urban population) + *β*(Log number of population).

Yt denoted the death rate; *β*0 was the intercept and *β* represented the association between each country-level sociodemographic covariate and the death rates of rotavirus infection; A positive value of *β* indicated a positive correlation, and a negative value indicated a negative correlation; We set the significance level at *P* < 0.05.

### Software

We used Microsoft Excel 2016 for data extraction, sorting, and cleaning, and R (version 3.2.3), SaTScan (version 9.5) and Join-point (version 4.8.0.1) for statistical analysis.

## Results

### Global burden and trends of rotavirus infection-associated deaths

Globally, rotavirus was the predominant pathogen causing diarrheal deaths among the 13 pathogens included in GBD 2019, accounting for 19.11% of deaths from diarrhea in 2019, corresponding to 235,331 deaths (110,221–415,457) (Additional file 1: Fig. S1**)**. In the past three decades, the number of deaths due to rotavirus infection decreased from 659,053 (95% UI 314,974–1,125,598) in 1990 to 235,331 (95% UI 110,221–415,457) in 2019 (Table 1). Regionally, the ASDRs decreased in most of the 21 regions, but increased in high-income North America from 1990 to 2019, with a significant AAPC (1.79%, *P* < 0.05) (Additional file 2: Fig. S2B). In the subgroup analysis by sex, the burden of rotavirus for males was higher than that for females in some regions, especially in Oceania. Interestingly, the ASDRs presented a significant uptrend for females in high-income North America (AAPC = 1.72%, *P* < 0.05) and Australia (AAPC = 0.74%, *P* < 0.05). Although the ASDRs of females in Western Europe also presented an uptrend, there was no statistical significance (AAPC = 0.16%, *P* = 0.66) (Additional file 2: Fig. S2).

**Fig. 1:**
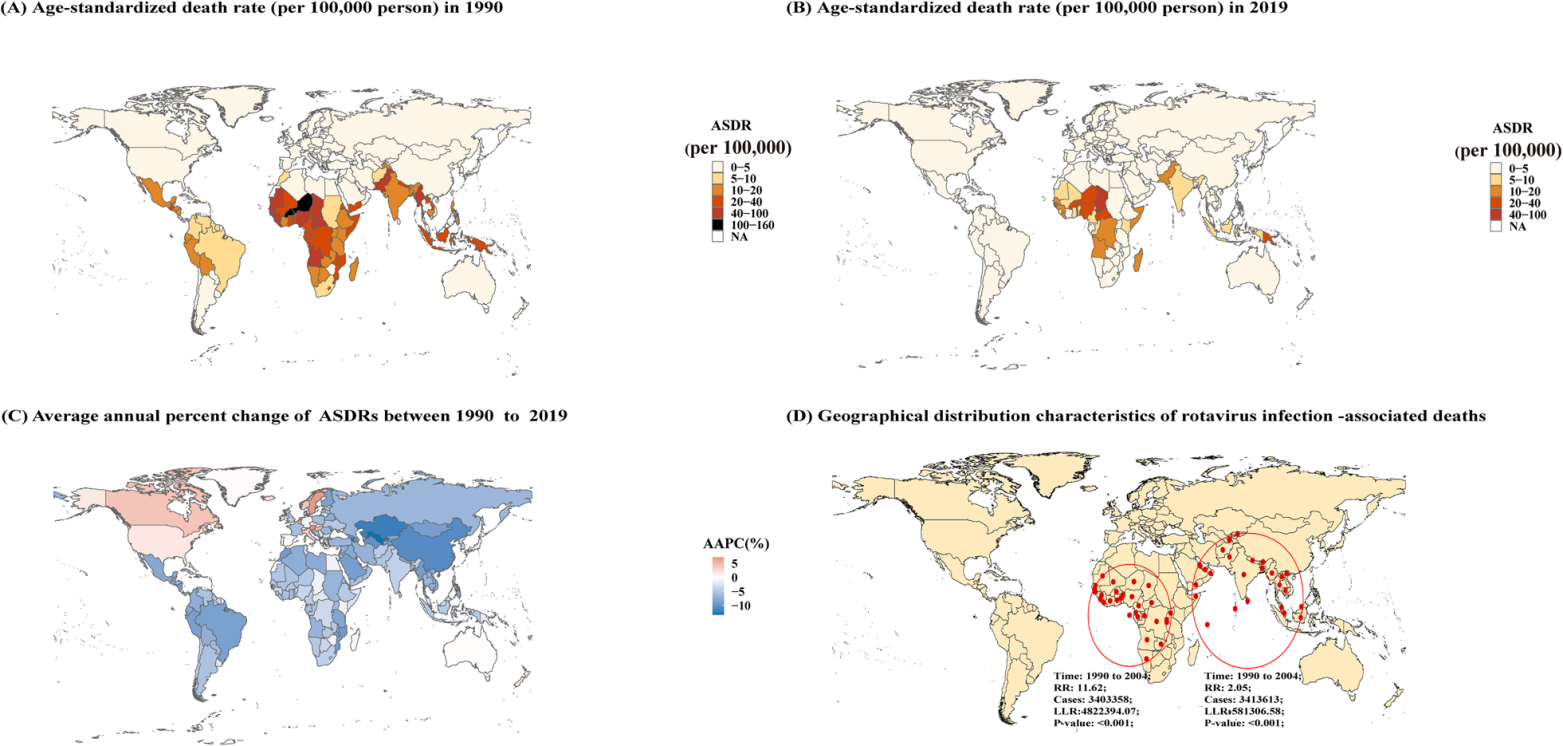
Global burden of rotavirus infection-associated deaths among 204 countries and territories. **A** ASDRs in 1990; **B** ASDRs in 2019; **C** AAPCs from 1990 to 2019; **D** Spatial and temporal aggregation from 1990 to 2019

Nationally, rotavirus caused a higher burden in African, Oceanian, and South Asian countries. In 1990, the ASDR of rotavirus infection was greatest in Niger, whereas in 2019, Chad showed the highest ASDR, followed by the Central African Republic and Niger. However, the lowest ASDR was recorded in Ireland in 1990, followed by Austria, whereas Estonia presented the lowest ASDR in 2019 (Fig. 1A–B). During the past 30 years, the ASDRs of rotavirus infection exhibited a downtrend in most countries, among which Uzbekistan presented the lowest AAPC (− 13.43%, *P* < 0.05). In contrast, the ASDRs presented an uptrend in countries such as Austria and Sweden, and the AAPC (7.13%, *P* < 0.05) in Austria was the greatest from 1990 to 2019 (Fig. 1C).

The spatial and temporal aggregation analysis showed two levels of spatial and temporal aggregation areas. The first level spatial and temporal aggregation area was composed primarily of 29 African countries such as Congo, Nigeria, and Chad. The gathering time was from 1990/1/1 to 2004/12/31, with a relative risk of 11.62 (LLR = 4,822,394.07, *P* < 0.05). The number of deaths due to rotavirus infection reported in the regions was 3,403,358, and the number of expected deaths was 378,326. The second-level spatial and temporal aggregation area was in South Asia, comprising 27 countries from 1990/1/1 to 2004/12/31, with a relative risk of 2.05 (LLR = 581,306.58, *P* < 0.05). The number of deaths due to rotavirus infection reported in the areas was 3,413,613, and the number of expected deaths was 1,906,684 (Fig. 1D).

### SDI- and age-specific burden and trends of rotavirus infection-associated deaths

**Fig. 2:**
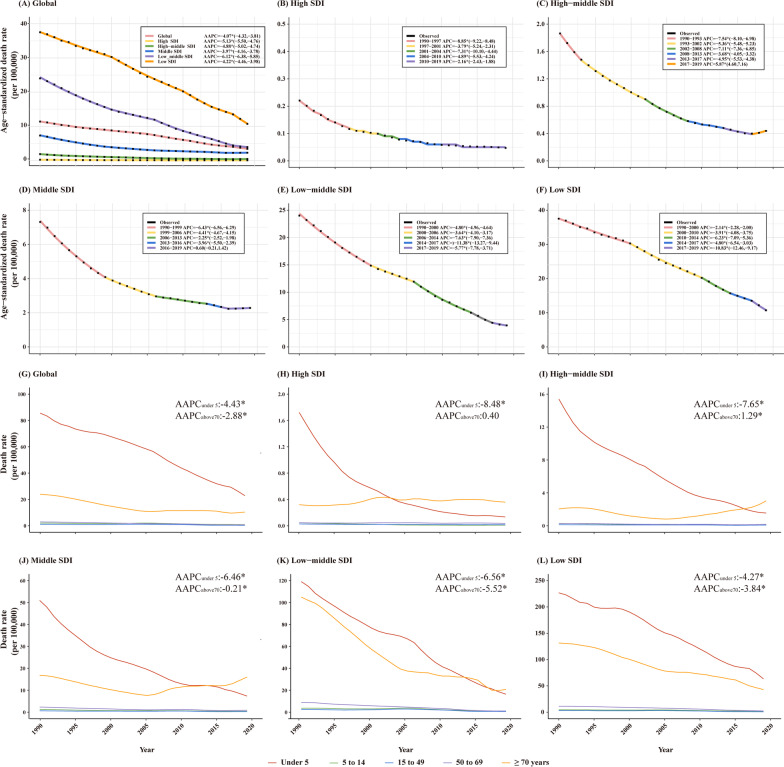
Age-specific temporal trends of rotavirus infection-associated deaths among SDI quintiles from 1990 to 2019. **A**–**F** Temporal trends of ASDRs globally and in different SDI countries over 30 years; **G**–**L** Age-specific temporal trends of death rates globally and in different SDI countries over 30 years. The APCs and AAPCs with asterisks (*) are statistically significant (*P* < 0.05)

Among the five SDI regions in 2019, the low SDI region bore the heaviest burden of rotavirus with a total number of deaths of 130,065 (95% UI 58,667–232,527) and an ASDR of 10.71 (95% UI 4.80–19.71) per 100,000 people (Table 1). From 1990 to 2019, the ASDRs presented a downtrend in five SDI regions with significant AAPCs (Fig. 2A–F). However, the ASDRs showed a significant uptrend in the high-middle SDI region from 2017 to 2019 (APC = 5.87%, *P* < 0.05). From 2016 to 2019, the ASDRs of rotavirus infection presented an uptrend in the middle SDI area with no statistical significance (APC = 0.60%, *P* = 0.14) (Fig. 2C, D). Furthermore, the ASDRs of rotavirus infection decreased with the increase of SDI index, presenting higher SDI regions showing a lower burden (Additional file 3: Fig. S3A).

**Fig. 3:**
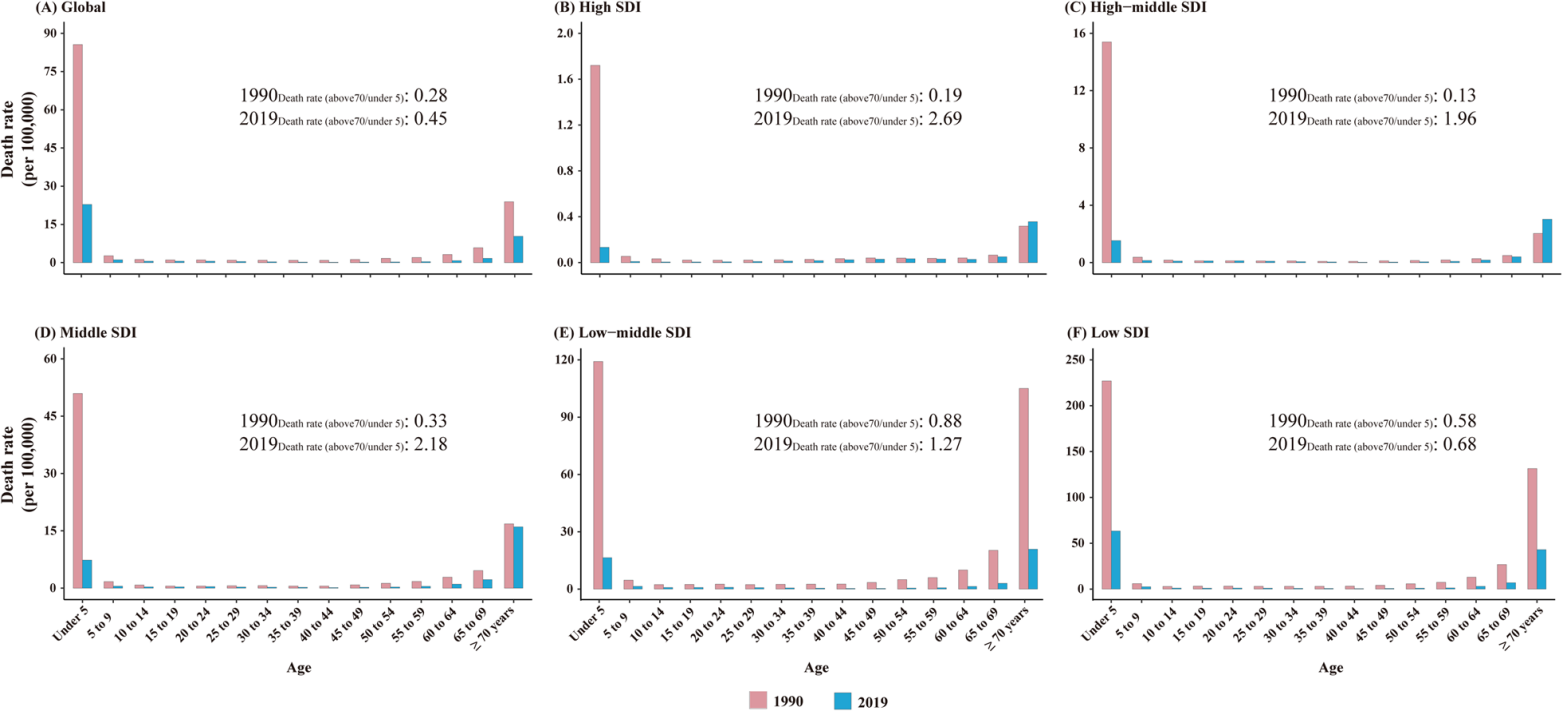
Age group distribution of the burden of rotavirus infection-associated deaths in 1990 and 2019 among SDI quintiles and worldwide. **A** Global; **B** High SDI; **C** High-middle SDI; **D** Middle SDI; **E** Low-middle SDI; **F** Low SDI

Children under 5 years and elderly individuals over 70 years were the two main age groups with significantly high death rates of rotavirus infection. The death rates of elderly individuals over 70 years showed an uptrend in the high-middle SDI area from 1990 to 2019 with a significant APC (1.29%, *P* < 0.05). In addition, the death rates of elderly individuals over 70 years also showed an uptrend in the high SDI region, though with no statistical significance (APC = 0.40%, *P* = 0.34) (Fig. 2H–L). Children under 5 years remained the main age group for rotavirus infection-associated deaths globally, with ratios (elderly individuals over 70 years vs. children under 5 years) from 0.28 in 1990 to 0.45 in 2019 (Fig. 3). Moreover, the death rates of elderly individuals over 70 years were found to be higher than those of children under 5 years in 2019 among high, high-middle, middle, and low-middle SDI regions, with the ratios (elderly individuals over 70 years vs. children under 5 years) from 1.27 in the low-middle SDI regions to 2.69 in the high SDI regions (Fig. 3). It meant that elderly individuals over 70 years necessitate attention in these regions.

### The effect of sociodemographic factors on the death rates of rotavirus infection

The GLM showed that the current health expenditure (% of GDP) (*β* = − 0.034, *P* = 0.049), GDP per capita (*β* = − 0.171, *P* = 0.040), and the number of physicians per 1000 people (*β* = − 0.285, *P* < 0.001) had significant negative effects on the death rates of rotavirus infection. The proportion of the urban population had a negative effect on the death rates with no statistical significance (*β* = − 0.003*, P* = 0.146). In contrast, the percentage of the population aged 0–14 (*β* = 0.178, *P* < 0.001) and the number of population (*β* = 0.108, *P* < 0.001) were significantly positively correlated with death rates. Although the current health expenditure per person (*β* = 0.108, *P* = 0.160) was also positively correlated with the death rates, there was no statistical significance (Table 2). A correlation analysis at the national level showed a significant negative correlation (R = − 0.64, *P* < 0.05) between the ASDRs of rotavirus infection and the SDI index in 2019 (Additional file 3: Fig. S3B).

## Discussion

This study estimated the global burden and trends of rotavirus infection-associated deaths in the past three decades. We showed that the ASDRs of rotavirus infection dramatically decreased from 1990 to 2019. However, the ASDRs remained unacceptably high in developing countries (e.g., Africa, Oceania, and South Asia), and the three countries with the highest ASDRs of rotavirus infection were all in Africa. Rotavirus is primarily transmitted via the fecal–oral route. In addition, the transmission may also occur through feces-contaminated food, water, hands and surfaces and respiratory droplets [6, 12]. Rotavirus infection mainly causes gastrointestinal symptoms such as diarrhea. Using rotavirus vaccine and enhanced interventions, such as improving sanitation and water sanitation and hygiene, supplementing vitamin A, zinc and oral rehydration salts (ORS), and appropriate case management, can reduce the burden of diarrhea [13, 14]. Compared to developed countries, the ASDRs of rotavirus infection were higher in developing countries, which might be attributed to lower hygiene standards, malnutrition, poor sanitation and inadequate access to medical care [8, 13, 15]. Vaccines are the most effective public health intervention for preventing and controlling infectious diseases. The use of vaccines reduced the hospitalization rates for rotavirus infection [16, 17]. Two oral vaccines, RotaTeq and Rotarix, have been successfully deployed since 2006, and rotavirus vaccines were introduced in more than 100 countries in 2019. Some studies have shown different efficacies of rotavirus vaccines in different countries [6, 18–20]. For example, Rotarix prevented 90.4% of severe rotavirus infections in Europe, but only 61.2% were prevented in Africa [21, 22]. RotaTeq’s efficacy in treating severe gastroenteritis ranges from 83 to 100% in developed countries such as the United States and Finland but only 30 to 74% in the developing country of Nicaragua [23, 24]. In addition, Chad and the Central African Republic, two countries in Africa with the highest ASDRs in 2019, had not yet introduced a rotavirus vaccine by the end of 2020 [25]. Therefore, there is a need to develop more effective vaccines and strengthen interventions. Countries that have not yet introduced rotavirus vaccines need to be encouraged to actively introduce vaccines to reduce the burden of rotavirus in these regions.

Differences in the burden of rotavirus infection-associated deaths have emerged between males and females. The ASDRs for males in some areas were higher than in females, particularly in Oceania, where the ratio of the ASDRs between males and females was 2.11. Salim et al. also reported that the ratio of rotavirus infection between boys and girls was 1.6: 1, indicating that boys are more susceptible to rotavirus infection than girls [26]. A previous study suggested that most infectious disease symptoms in children are more likely to occur in males [27]. In addition, sex differences in the burden of rotavirus infection-associated deaths can be explained by various factors, such as differences in behavior, hormonal differences, immune function differences, and gene expression between males and females [28, 29]. All these factors could lead to differences in the burden of rotavirus infection-associated deaths between the sexes. However, the exact biological mechanisms involved remain unclear and further research is needed.

Children under 5 years and elderly individuals over 70 years were the two main age groups with a significantly high burden of rotavirus, as they have relatively poor immunity and are more susceptible to infectious diseases. This agrees with the finding that the burden of diarrheal diseases was heaviest in these two age groups [1]. Because global initiatives to reduce the burden of diarrhea have mostly focused on children under 5 years, and the use of rotavirus vaccines, the death rates of rotavirus infection among children under 5 years have declined dramatically in the past three decades. In 2019, the death rates of elderly individuals over 70 years were higher than those of children under 5 years in high, high-middle, middle and low-middle SDI regions. This might be ascribed to the rapid aging of populations in both developed and developing countries, and elderly individuals are vulnerable to death from infectious diseases because of weakened immunity and underlying diseases [30]. Population aging is a global problem, and studies have reported that population growth and aging lead to increased disease burden [31, 32]. Moreover, more elderly individuals live in hospitals and nursing homes in higher SDI regions with improved health and medical security. Edmonson et al. reported that when rotavirus infection occurs in nursing homes, it spreads quickly [33]. In addition, the increased burden of rotavirus among elderly individuals might also be due to increased pathogen testing in health care facilities of stool samples from elderly patients with gastroenteritis [34, 35]. This finding emphasizes the need for targeted interventions for rotavirus infections in elderly individuals over 70 years, such as whether vaccines should be recommended for these individuals.

Notably, our findings also indicated that the burden of rotavirus was negatively correlated with current health expenditure (% of GDP), GDP per capita, and the number of physicians per 1000 people. Claudine et al. reported that children from low-income families were at higher risk of diarrhea than those from wealthy families [36]. Previous studies also reported that risk factors associated with diarrheal mortality are inadequate access to medical care, malnutrition and poverty [14, 37, 38], suggesting that increasing GDP per capita and health care levels (health expenditure and the number of physicians) can reduce the burden of rotavirus-associated diarrhea. There was a positive correlation between the percentage of the population aged 0–14 and the burden of rotavirus. This is easy to explain because children under 5 years old were most at risk for rotavirus due to their weakened immune systems. The above risk factors can support the implementation of specific policies to reduce the burden of rotavirus.

### Limitation

First, although the sensitivity and specificity of ELISA are high (> 95%) and the detection of rotavirus has been increasingly implicated in diarrheal diseases, targeted pathogen detection of rotavirus is still insufficient in diarrhea cases. In adults, diarrheal diseases attributable to rotavirus are mostly subclinical or mild, they are less likely to go to the hospital, and hospitals rarely conduct rotavirus testing in adult patients with diarrhea. As a result, the number of confirmed rotavirus cases reported to public health authorities may be lower than is the case. In particular, in developing regions, data on the incidence of diarrhea and its causes are scarce, and this area relies more on model estimation. All these factors may lead to an underestimation of the number and morbidity of rotavirus infections. Second, our study discussed only the burden of rotavirus infection-associated death, which only partly reflects the disease burden of rotavirus. In the future, more research is needed to explore the disease burden of rotavirus with multiple indicators to provide more comprehensive evidence and support for policy-making**.**

## Conclusions

We analyzed the global burden and trends of rotavirus infection-associated deaths in the past three decades by age and sex in different regions and countries. Although the global trend has decreased substantially in the past three decades, the burden of rotavirus remained high in Africa, Oceania, and South Asia. Children under 5 years and elderly individuals over 70 years population were most at risk of rotavirus infection-associated deaths, especially for elderly individuals over 70 years in relatively high SDI regions. More attention should be paid to these regions and populations, and effective public health policies should be implemented, including improving medical care, strengthening the protection of vulnerable groups, increasing the coverage of existing vaccines, and developing more effective vaccines in the future.

**Table 1:** The death numbers and ASDR of rotavirus infection in 1990 and 2019 with AAPCs over the 30 years

	1990		2019		
	Death number(95% UI)	ASDR(95% UI)	Death number(95% UI)	ASDR(95% UI)	AAPC, %(95% CI)
Global	659,053 (314,974–1,125,598)	11.39 (5.46–19.48)	235,331 (110,221–415,457)	3.41 (1.60–6.01)	− 4.07* (− 4.32,− 3.81)
*Gender*					
Male	352,867 (166,183–608,843)	12.65 (6.01–21.86)	122,824 (59,570–215,761)	3.65 (1.78–6.42)	− 4.18* (− 4.30,− 4.07)
Female	306,187 (145,000–534,575)	10.55 (4.85–18.75)	112,506 (50,821–208,725)	3.21 (1.46–5.83)	− 4.03* (− 4.25,− 3.80)
*SDI rank*					
High SDI	1443 (674–2549)	0.22 (0.10–0.39)	704 (278–1444)	0.05 (0.02–0.09)	− 5.13* (− 5.50,− 4.76)
High-middle SDI	18,990 (9183–30,411	1.86 (0.90–2.99)	6201 (2951–11,537)	0.44 (0.22–0.75)	− 4.88* (− 5.02,− 4.74)
Middle-SDI	126,931 (61,348–209,772)	7.31 (3.47–12.34)	42,881 (21,626–76,459	2.28 (1.17–4.02)	− 3.97* (− 4.16,− 3.78)
Low-middle SDI	260,739 (119,730–467,415)	24.01 (11.32–42.68)	55,338 (25,198–101,806)	3.94 (1.73–7.54)	− 6.12* (− 6.38,− 5.85)
Low SDI	250,593 (113,581–428,636)	37.52 (17.2–67.01)	130,065 (58,667–232,527)	10.71 (4.80–19.71)	− 4.22* (− 4.46,− 3.98)
*GBD regions*					
Andean Latin America	6002 (3725–8524)	13.64 (8.29–20.36)	757 (369–1465)	1.30 (0.62–2.55)	− 8.22* (− 8.59,− 7.85)
Australasia	9 (4–16)	0.04 (0.02–0.08)	25 (9–53)	0.05 (0.02–0.11)	0.63 (− 0.01,1.27)
Caribbean	3603 (1701–6255)	9.07 (4.3–15.79)	763 (292–1481)	1.75 (0.65–3.48)	− 6.04* (− 6.49,− 5.59)
Central Asia	1910 (716–3917)	2.03 (0.76–4.16)	135 (46–302)	0.14 (0.05–0.32)	− 8.70* (− 9.01,− 8.39)
Central Europe	262 (125–429)	0.31 (0.15–0.51)	27 (14–47)	0.04 (0.02–0.06)	− 6.82* (− 7.28,− 6.37)
Central Latin America	21,355 (11,627–32,566)	11.96 (6.42–18.51)	2765 (1369–4729)	1.23 (0.61–2.11)	− 7.51* (− 7.72,− 7.30)
Central Sub-Saharan Africa	35,801 (16,072–62,837)	46.14 (21.48–80.74)	15,409 (5216–32,211)	11.99 (4.53–23.29)	− 4.41* (− 4.80,− 4.02)
East Asia	39,195 (18,103–61,488)	3.44 (1.63–5.43)	1227 (626–2020)	0.14 (0.07–0.22)	− 10.51* (− 11.04,− 9.98)
Eastern Europe	618 (298–963)	0.40 (0.19–0.62)	71 (33–117)	0.06 (0.03–0.09)	− 6.35* (− 7.27,− 5.43)
Eastern Sub-Saharan Africa	67,508 (30,011–121,444)	20.49 (9.16–37.71)	28,148 (13,311–50,067)	4.95 (2.33–9.18)	− 4.76* (− 5.00,− 4.51)
High-income Asia Pacific	179 (76–336)	0.13 (0.06–0.24)	289 (103–647)	0.07 (0.03–0.14)	− 2.19* (− 2.63,− 1.75)
High-income North America	45 (22–76)	0.02 (0.01–0.03)	130 (51–274)	0.03 (0.01–0.06)	1.79* (1.58,2.01)
North Africa and Middle East	26,666 (10,860–50,417)	5.09 (2.10–9.67)	7974 (3394–14,796)	1.36 (0.58–2.54)	− 4.45* (− 4.80,− 4.11)
Oceania	1028 (495–1725)	32.23 (16.62–57.66)	1379 (676–2384)	18.38 (8.90–32.64)	− 1.93* (− 2.15,− 1.71)
South Asia	191,715 (83,315–357,226)	25.42 (11.10–46.54)	64,372 (28,290–126,945)	5.77 (2.47–11.49)	− 5.00* (− 5.36,− 4.64)
Southeast Asia	81,417 (36,466–141,759)	16.23 (7.09–28.74)	13,618 (6039–25,555)	2.52 (1.11–4.82)	− 6.22* (− 6.33,− 6.11)
Southern Latin America	320 (140–588)	0.66 (0.29–1.23)	117 (46–232)	0.16 (0.07–0.32)	− 4.62* (− 5.53,− 3.70)
Southern Sub-Saharan Africa	4478 (1730–9078)	7.71 (2.93–15.25)	1687 (652–3543)	2.63 (0.97–5.72)	− 3.67* (− 4.14,− 3.21)
Tropical Latin America	10,271 (4282–19,778)	6.37 (2.67–12.09)	911 (389–1756)	0.48 (0.20–0.94)	− 8.65* (− 9.47,− 7.83)
Western Europe	157 (61–314)	0.03 (0.01–0.07)	262 (104–533)	0.03 (0.01–0.06)	− 0.77* (− 1.36,− 0.18)
Western Sub-Saharan Africa	166,514 (72,971–296,750)	59.94 (26.39–106.39)	95,265 (41,399–172,892)	18.26 (8.15–32.78)	− 4.07* (− 4.32,− 3.81)

The AAPCs with asterisks (*) are statistically significant (*P* < 0.05).

*ASDR* age-standardized death rate; *AAPC* average annual percentage change.

**Table 2:** The correlation analysis of sociodemographic factors to the death rates of rotavirus infection

Factors	*β*	Std. Error	*P *value
Current health expenditure (% of GDP)	− 0.034	0.017	0.049
Log current health expenditure per person	0.108	0.077	0.160
Percentage of the population aged 0–14	0.178	0.004	< 0.001
Log GDP per capita	− 0.171	0.083	0.040
Number of physicians per 1000 people	− 0.285	0.028	< 0.001
Proportion of the urban population	− 0.003	0.002	0.146
Log number of population	0.108	0.013	< 0.001
(Intercept)	− 5.673	0.524	< 0.001

## Supplementary Information

**Additional file 1.** **Fig. S1**. 13 pathogens caused diarrhea globally for 1990 and 2019, including deaths number and their percentage.

**Additional file 2. Fig. S2**. Temporal trends and gender-specific burden of rotavirus infection-associated deaths in 21 regions. **A** ASDRs in 1990; **B** AAPCs from 1990 to 2019; **C** ASDRs in 2019. The AAPCs with asterisks (*) are not statistically significant (*P* > 0.05).

**Additional file 3.** **Fig. S3**. Association between rotavirus infection-associated deaths and SDI. **A** ASDRs of 21 regions; **B** ASDRs of 204 countries and territories.

## Data Availability

All data generated or analysed during this study are included in this published article [and its supplementary information files].

## Abbreviations

GBD 2019: Global Burden of Disease 2019 Study
ASDR: Age-standardized death rate
SDI: Sociodemographic index
95% UI: 95% uncertainty interval
AAPC: Average annual percentage change
WHO: World Health Organization
EuroRotaNet: European Rotavirus Network
APC: Annual percentage change
CI: Confidence intervals
LLR: Logarithmic likelihood ratio
RR: Relative risk
GDP: Gross domestic product
GLM: Generalized linear model
ORS: Oral rehydration salts
