# Supplementary figures and images for: Global burden and trends of rotavirus infection-associated deaths from 1990 to 2019: an observational trend study
Source: Virol J. 2022 Oct 20;19:166. doi: 10.1186/s12985-022-01898-9 (PMC9585833; doi:10.1186/s12985-022-01898-9)

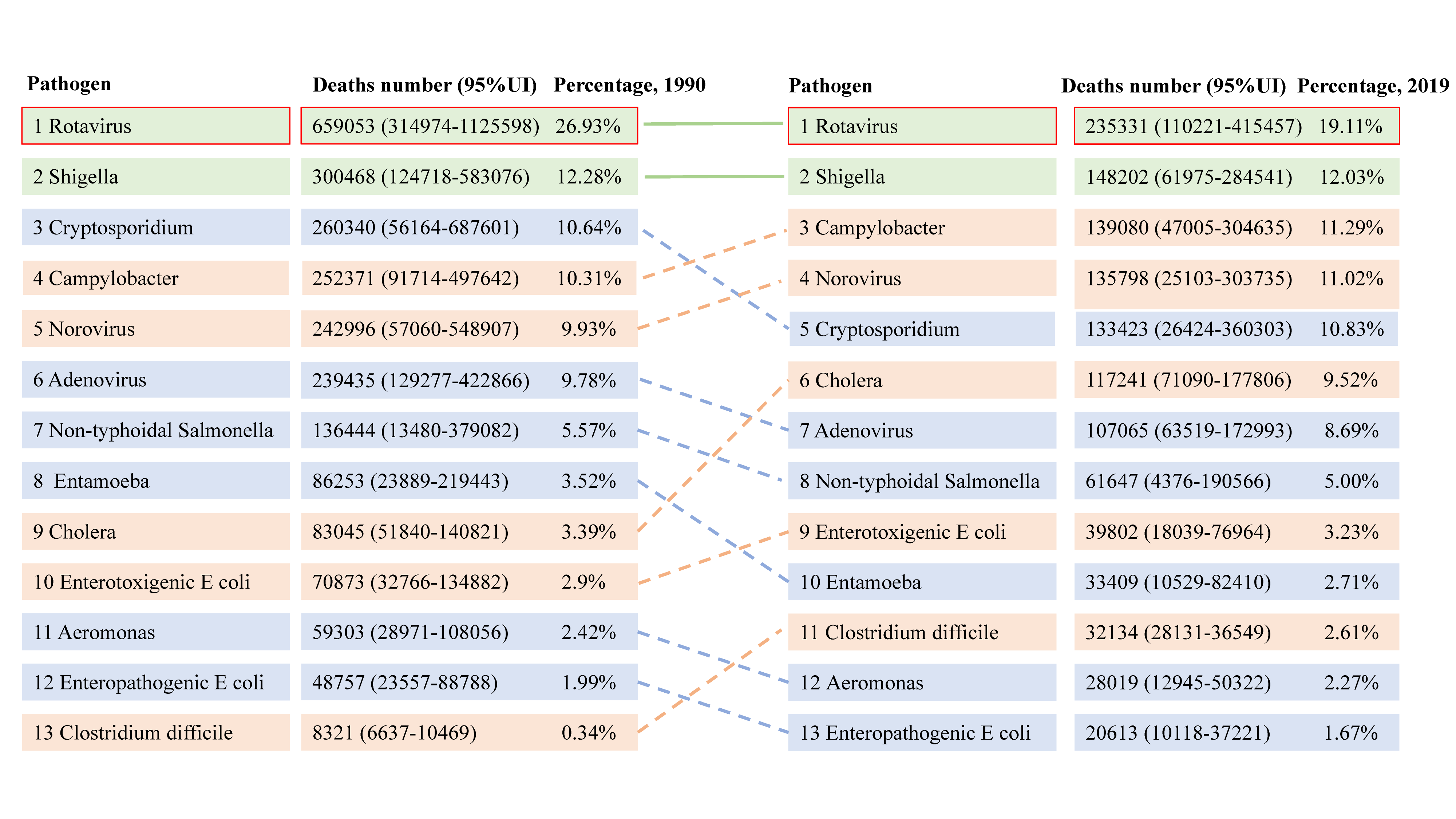

Supplement: Supplementary file 1 — Additional file 1. Fig. S1. 13 pathogens caused diarrhea globally for 1990 and 2019, including deaths number and their percentage. [file 12985_2022_1898_MOESM1_ESM.tif]

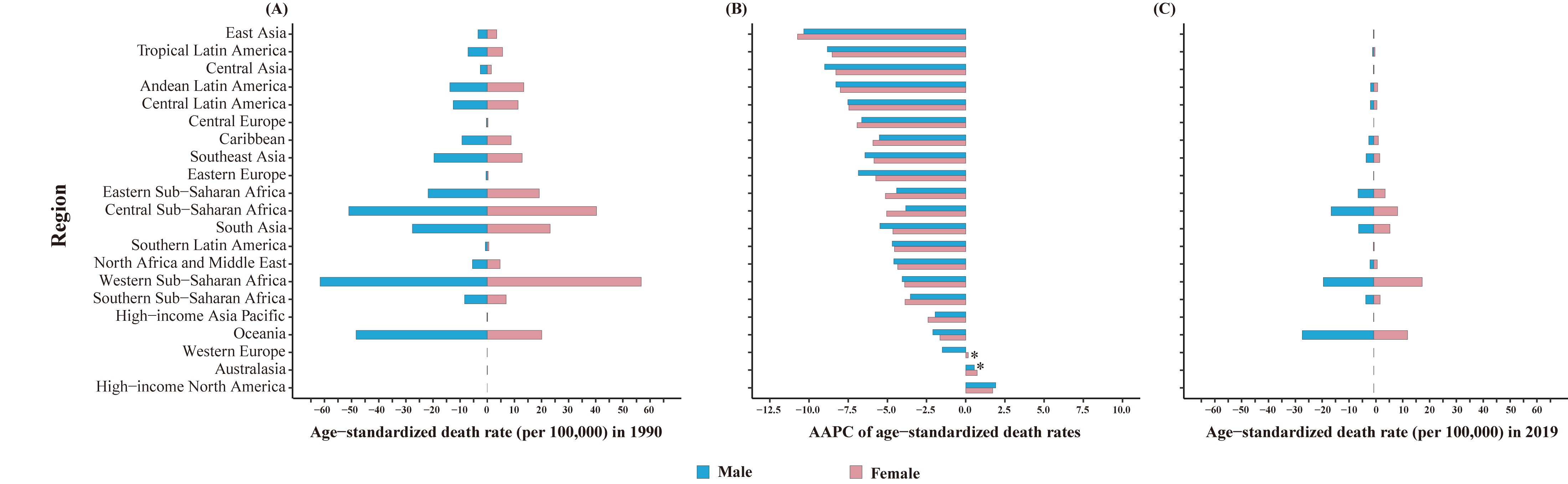

Supplement: Supplementary file 2 — Additional file 2. Fig. S2. Temporal trends and gender-specific burden of rotavirus infection-associated deaths in 21 regions. A ASDRs in 1990; B AAPCs from 1990 to 2019; C ASDRs in 2019. The AAPCs with asterisks (*) are not statistically significant (P > 0.05). [file 12985_2022_1898_MOESM2_ESM.tif]

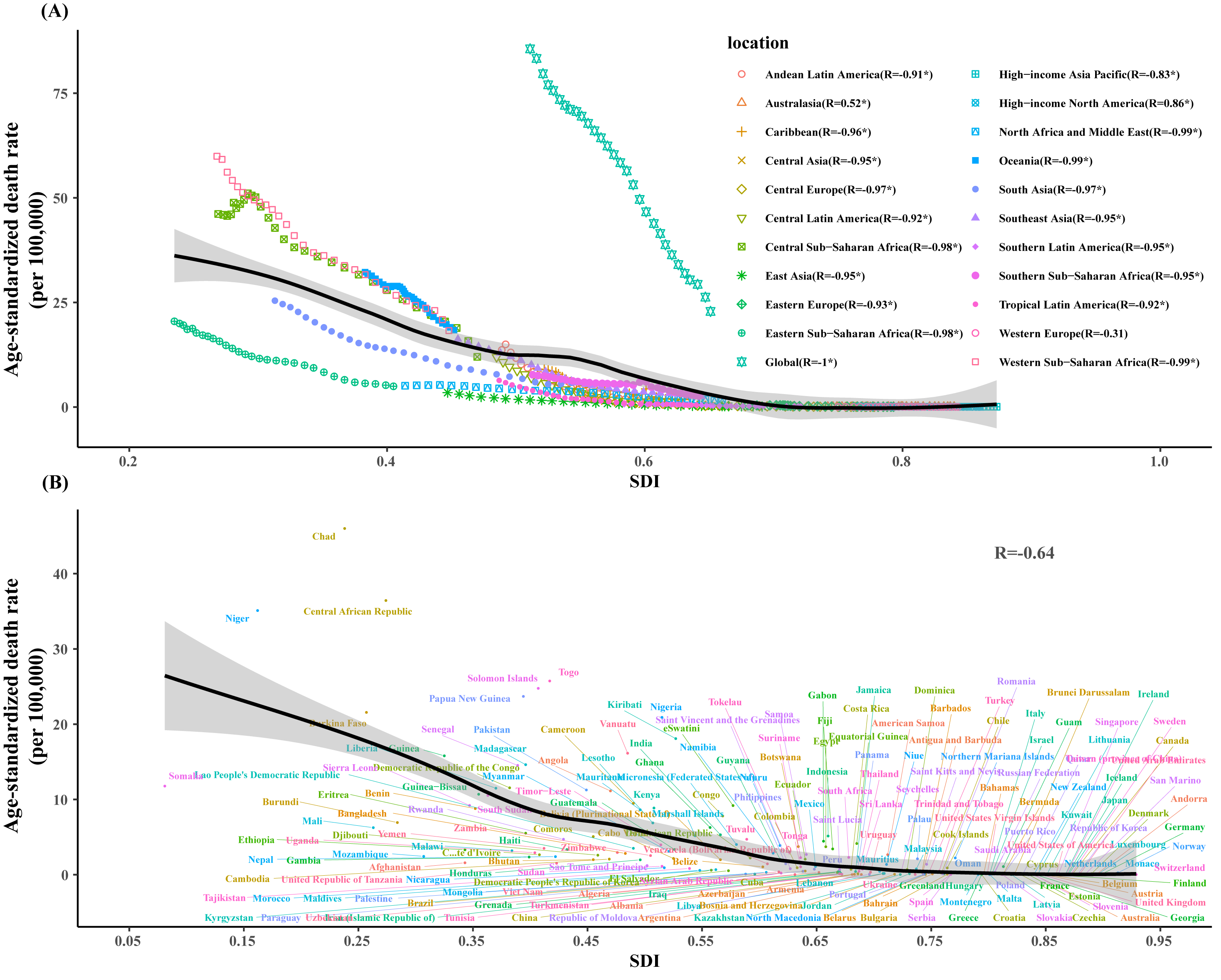

Supplement: Supplementary file 3 — Additional file 3. Fig. S3. Association between rotavirus infection-associated deaths and SDI. A ASDRs of 21 regions; B ASDRs of 204 countries and territories. [file 12985_2022_1898_MOESM3_ESM.tif]
